# Low pectoralis muscle index, cavitary nodule or mass and segmental to lobar consolidation as predictors of primary multidrug-resistant tuberculosis: A comparison with primary drug sensitive tuberculosis

**DOI:** 10.1371/journal.pone.0239431

**Published:** 2020-10-05

**Authors:** Hwa Seon Shin, Dae Seob Choi, Jae Boem Na, Hye Young Choi, Ji-Eun Kim, Ho Cheol Choi, Jung Ho Won, Seung Jun Lee, Mi Jung Park

**Affiliations:** 1 Department of Radiology, Gyeongsang National University School of Medicine and Gyeongsang National University Hospital, Jinju, Gyeongsangnam-do, Republic of Korea; 2 Division of Pulmonology, Department of Internal Medicine, Gyeongsang National University School of Medicine and Gyeongsang National University Hospital, Jinju, Gyeongsangnam-do, Republic of Korea; Clinic for Infectious and tropical diseases, Clinical centre of Serbia, SERBIA

## Abstract

**Background:**

The loss of muscle mass in primary multidrug-resistant tuberculosis (MDR-TB) has not been examined in previous studies. This study aimed to investigate that low pectoralis muscle index and characteristic CT features can help differentiate patients with primary MDR-TB from those with drug-sensitive tuberculosis (DS-TB).

**Material and methods:**

From 2010 to 2016, we retrospectively enrolled 90 patients with primary MDR-TB and 90 age- and sex-matched patients with primary DS-TB. The pectoralis muscle mass was quantitatively measured on axial CT images using density histogram analysis. The pectoralis muscle index (PMI) was defined as the pectoralis muscle mass divided by body mass index. We compared the PMI and characteristic CT features of pulmonary tuberculosis between the two groups.

**Results:**

Low PMI, segmental to lobar consolidation, cavity in consolidation, cavitary nodule or mass, and bilateral involvement were more frequently observed in patients with MDR-TB than in those with DS-TB. In stepwise multivariate logistic regression analysis, low PMI (odds ratio, 2.776; 95% confidence interval, 1.450–5.314; p = 0.002), segmental or lobar consolidation (odds ratio, 3.123; 95% confidence interval, 1.629–5.987; p = 0.001), and cavitary nodule or mass (odds ratio, 2.790; 95% confidence interval, 1.348–5.176; p = 0.002) were significant factors for MDR-TB.

**Conclusion:**

Low pectoralis muscle index, segmental to lobar consolidation and cavitary nodule or mass can help differentiate primary MDR-TB from DS-TB.

## Introduction

Tuberculosis (TB) is a deadly infectious disease worldwide. The WHO estimates that over 10 million people developed TB and 1.5 million people died from TB worldwide in 2018 [1]. In particular, multidrug-resistant TB (MDR-TB) remains a public health concern because the treatment failure rate is high, regardless of long treatment period. MDR-TB refers to tuberculous infection caused by acid-fast bacterial organisms resistant to at least two anti-tuberculous medications, including isoniazid and rifampin. In 2018, the WHO estimated that there were approximately 377,520 new cases of MDR-TB [1]. Globally, 3.4% of new TB cases and 18% of patients previously treated with anti-tuberculous medication had MDR-TB or rifampicin-resistant TB [1].

TB is divided into primary TB and acquired TB according to the history of anti-tuberculous treatment. Primary anti-tuberculous drug resistance indicates that the patient has no anti-tuberculous treatment or a previous treatment history of less than 1 month. In contrast, acquired anti-tuberculous drug resistance indicates that the patient has a previous treatment history of more than 1 month. It is important to detect MDR-TB early to avoid ineffective first-line TB treatment and determine other proper medications. A history of previous TB and intermittent and short-term TB medication use are crucial factors for acquired MDR-TB. However, primary MDR-TB is more difficult to detect than acquired MDR-TB.

Malnutrition is a risk factor of active status [2], disease severity [3] and relapse [4] of TB. Malnutrition is caused by the increased metabolic demands of chronic inflammatory responses related to TB [5]. Body mass index (BMI) has been used as an easy and comprehensible tool to estimate nutritional status, but it has some limitations in representing body composition. The body composition of each patient can be different, regardless of weight or BMI. The gold standard method of body composition analysis is dual-energy X-ray absorptiometry, but this method takes time to measure body composition. Chest CT has been widely used to evaluate the active status, disease severity and treatment response of TB [6]. Furthermore, CT can differentiate between muscle and fat and quantify muscle mass. The pectoralis muscle mass can be a useful indicator to assess the skeletal muscle area without additional radiation exposure. Several studies have reported that low pectoralis muscle mass on chest CT is associated with chronic diseases such as malignancy [7,8] and COPD [9]. Previous studies found that muscle wasting was prolonged in the follow-up period ranging from 6 months to 2 years after TB treatment [5,10,11]. Other reports have suggested that patients with weight loss were prone to developing MDR-TB [12,13]. We speculated that low muscle mass is more severe in patients with primary MDR-TB than in patients with primary DS-TB. The purpose of this study is to assess that low pectoralis muscle mass and pulmonary findings on chest CT can be useful indicators to predict primary MDR-TB from primary DS-TB for selection of proper anti-tuberculous treatment.

## Materials and methods

### Patients

This study was conducted as a retrospective matched, case-control study and included 2181 pulmonary tuberculosis patients who received treatment in the Gyeongsang National University Hospital from January 2010 to December 2016. We classified the tuberculosis patients into two groups, MDR-TB and DS-TB. MDR-TB was defined as TB from a strain resistant to at least both isoniazid and rifampin. DS-TB was defined as TB with a drug sensitivity to all four first-line anti-TB drugs, isoniazid, rifampicin, ethambutol, and streptomycin.

The inclusion criteria for patients with primary pulmonary tuberculosis were as follows: (1) age ≥20 years old, (2) a diagnosis of pulmonary tuberculosis, (3) positive culture for mycobacterium tuberculosis on bronchoscopy within 1 month of pulmonary TB diagnosis, (4) available drug sensitivity test results, and (5) chest CT within 1 month of pulmonary TB diagnosis. The exclusion criteria for patients with primary pulmonary TB were as follows: (1) acquired TB, (2) insufficient data for the diagnosis or treatment of pulmonary TB, (3) mixed infection with nontuberculous mycobacterium, (4) malignancy, and (5) interstitial lung disease. All patients were HIV seronegative.

Among the 473 patients with MDR-TB, we excluded patients with acquired MDR-TB (n = 352), no available data on the TB diagnosis and management (n = 3), mixed infection with nontuberculous mycobacterium (n = 5), malignancy (n = 19), and interstitial lung disease (n = 4). Among the 1708 patients with DS-TB, we excluded patients with acquired DS-TB, and 90 patients were enrolled as controls. Muscle mass can be affected by age and sex [14]; thus, we enrolled age- and sex-matched patients with DS-TB as controls. The controls were matched by sex and age within 2 years. The Institutional Review Board of our hospital approved this study (Gyeongsang National University Hospital-2019-06-015, and the requirement for informed consent was waived because our study was an observational retrospective study.

### Image acquisition

Chest CT examinations were performed using a 64-detector CT (Brilliance-64; Philips Medical Systems, The Netherlands). CT was performed with a detector configuration of 64 x 0.625 mm, a tube voltage of 120 kVp, a fixed tube current of 200 mAs, a pitch of 0.923, a gantry rotation time of 0.5 seconds, and a smooth reconstruction filter (Philips “B” filter). The attenuation coefficients ranged from -1024 to 3072 Hounsfield units (HU).

All patients were examined during full inspiration with their arm raised and in the supine position. Chest CT scans were performed from the lung apex to the diaphragm in the cranio‐caudal direction. No contrast material was applied in this study.

### Image analysis

We obtained axial images using a high-spatial-frequency algorithm with a 1-mm slice thickness and 1-mm intervals and for lung parenchyma analysis. Two chest radiologists reviewed the thin-section chest CT images. The radiologists were blinded to all clinical information except that the patients had been diagnosed with pulmonary tuberculosis. A final decision about these findings was reached by consensus.

We reviewed the axial images in both mediastinal (window width, 400 Hounsfield units [HU]; window level, 25 HU) and lung (window width, 1500 HU; window level, -700 HU) settings. The observers retrospectively interpreted the axial images for centrilobular nodules (including a tree-in-bud pattern), large nodules (nodules 10–30 mm in diameter), consolidation (further divided into lobular or subsegmental and segmental or lobar), cavity (further divided into cavity in consolidation and cavitary nodule or mass), fibrotic scar, bronchiectasis, calcifications, pleural or pericardial effusion, lymphadenopathy (lymph node enlargement of more than 1 cm in the mediastinum or hilum) and density of lymph node (calcified or noncalcified). In addition, lobular consolidation was defined as the presence of a consolidative lesion 0.5 to 3.0 cm in size that was polygonal in shape with a subpleural location. Laterality (unilateral or bilateral) of the lung parenchymal lesions was also analyzed. Six locations (right upper lobe, right middle lobe, right lower lobe, left upper lobe except the lingular segment, lingular segment, and left lower lobe) were used to describe the extent of the lung lesions.

**Fig 1:**
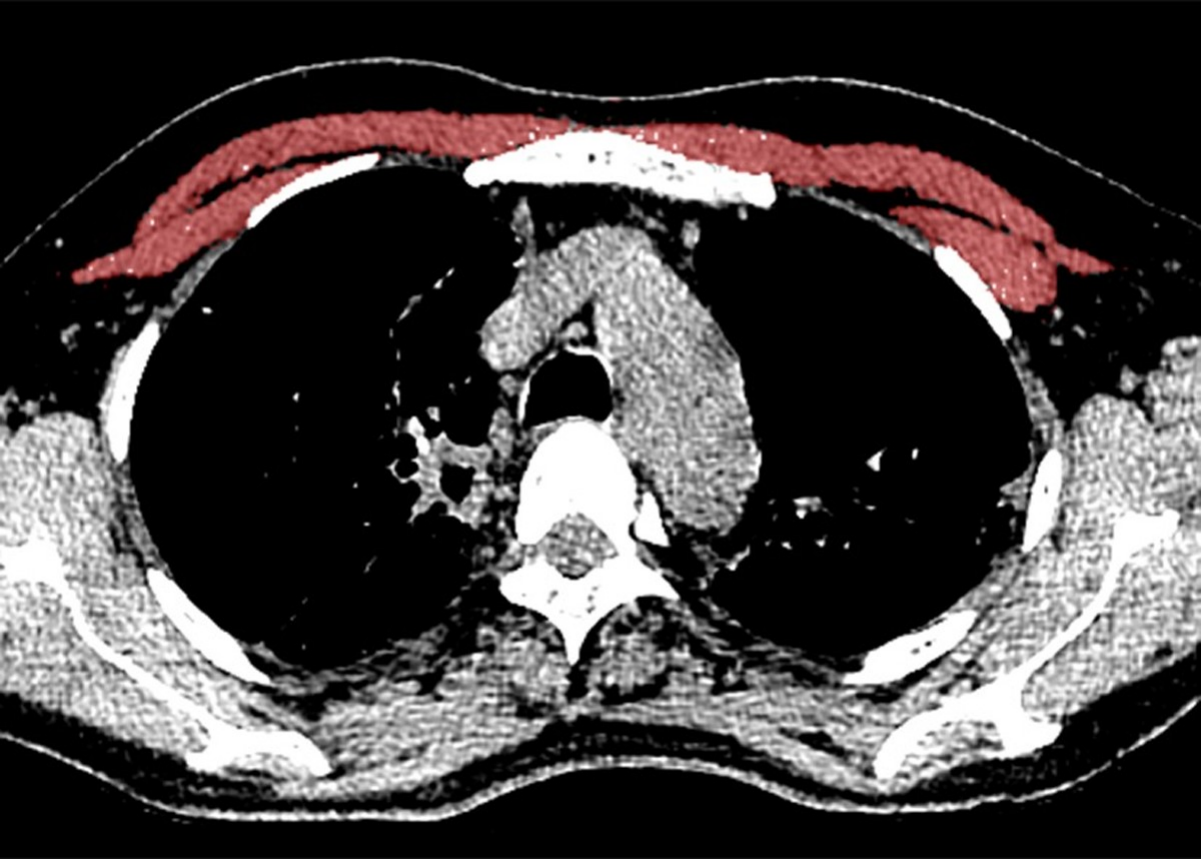
Measurement of pectoralis muscle area on chest CT. The pectoralis muscle area was quantitatively measured in the axial CT (3mm slice thickness) with standard reconstruction algorithm and mediastinal setting. The bilateral pectoralis muscle area (pectoralis major and minor) shaded in purple.

The axial images were reconstructed using a standard reconstruction algorithm with a slice thickness of 3 mm and slice interval of 3 mm to measure the pectoralis muscle mass. The mass of the pectoralis muscle, including the pectoralis major and minor, was measured by one radiologist with 10 years of experience. We used open-source program (Slicer 4.8.1, http://www.slicer.org as described previously [15] and the measurement method was as follows. First, reconstructed axial images were analyzed at the level of the fourth thoracic vertebra. Second, a region of interest was placed freehand around the outermost border of the muscles. Third, the area of the pectoralis muscles visualized from -29 to 100 HU was calculated using CT histogram analysis (Fig 1). The bilateral pectoralis muscles were measured separately, and the two values were averaged. The pectoralis muscle mass area was divided by body mass index (BMI), and we reported these values as the pectoralis muscle index (PMI).

### Statistical analysis

Statistical analyses were performed using SPSS 21.0 software (SPSS Inc., Chicago, IL, USA). The clinical features and presence of each pattern of parenchymal abnormality in primary MDR-TB and DS-TB were evaluated using the chi-square test. The number of involved lobes with lung parenchymal abnormalities was compared using the Mann-Whitney U test. Potentially significant variables (P<0.10) in univariate analyses were included in a multivariate analysis. Stepwise logistic regression analysis was performed to determine if the CT features and PMI could distinguish between primary MDR-TB and DS-TB. The median PMI value was 68.2 and considered the cutoff value. A p value less than 0.05 was considered statistically significant.

## Results

The characteristics of the patients with primary MDR-TB and DS-TB are summarized in Table 1. The mean age, sex ratio and body mass index were not significantly different between patients with MDR-TB and those with DS-TB. The prevalence of symptoms and disease duration were not significantly different between the two groups.

**Table 1:** Clinical features of patients with primary MDR-TB and patients with DS-TB.

Characteristics	Primary MDR-TB (n = 90)	Primary DS-TB (n = 90)	p value
Age (years)	48.9 ± 14.0	48.8 ± 12.9	0.947
Sex (male)	21 (28.4%)	21 (28.4%)	1.000
Body mass index (kg/m^2^)	21.8 ± 2.9	21.2 ± 2.6	0.132
Diabetes	21 (23.3%)	18 (20.0%)	0.587
Hypertension	13 (14.4%)	15 (17.8%)	0.543
Fever	11 (12.2%)	12 (13.3%)	0.823
Cough	74 (82.2%)	73 (81.1%)	0.847
Hemoptysis	7 (7.8%)	10 (11.1%)	0.445
Dyspnea	13 (14.4%)	5 (5.6%)	0.050
Chest pain	4 (4.4%)	8 (8.9%)	0.232
Duration of illness (days)	36.0 ± 46.2	40.1 ± 63.8	0.620
Sputum AFB smear positive	50 (55.6%)	38 (42.2%)	0.074
Extrapulmonary tuberculosis	5 (5.6%)	6 (6.7%)	0.756

AFB. Acid- Fast Bacilli; DS-TB, drug sensitive tuberculosis; MDR-TB, multidrug resistant tuberculosis.

**Fig 2:**
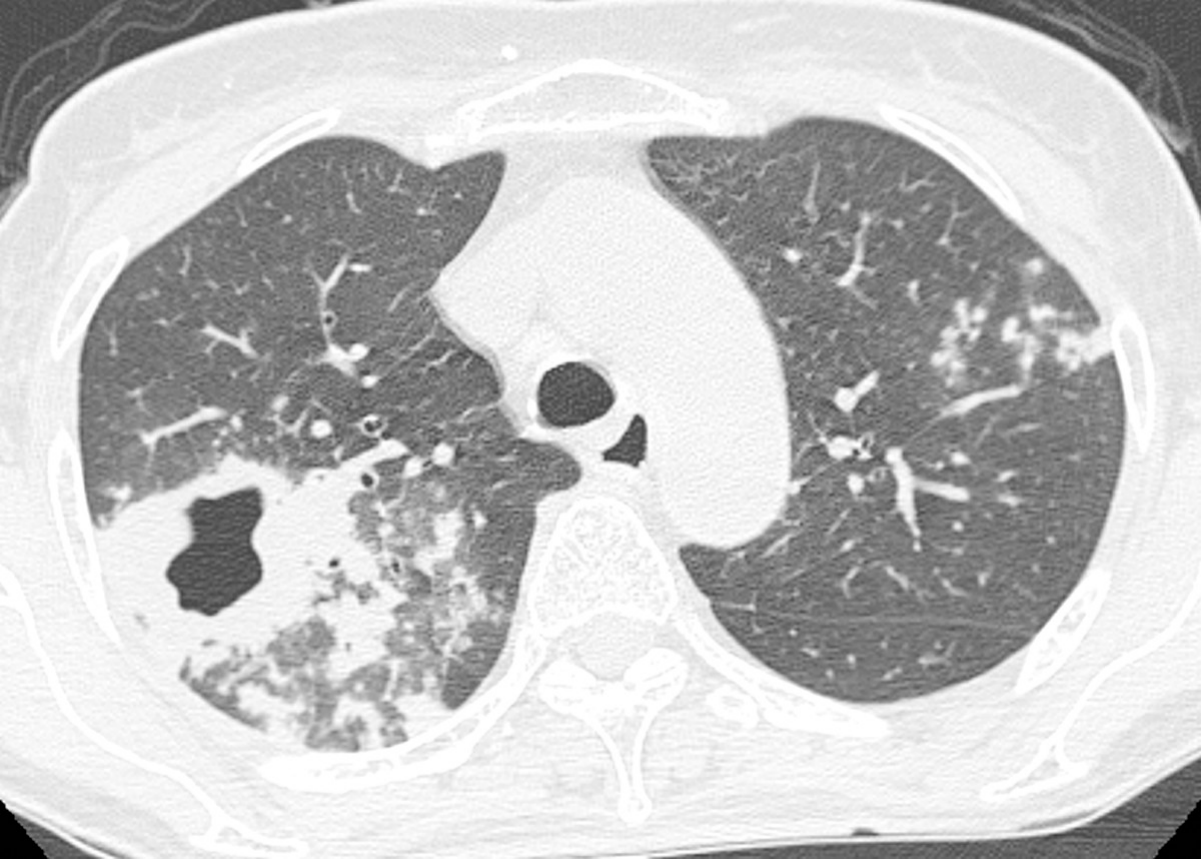
Primary multidrug-resistant tuberculosis with 37-year-old woman. The thin section CT (1mm slice thickness) with high-spatial-frequency algorithm and lung window setting shows thick wall cavity and subsegmental or lobular consolidation in right upper lobe. Tree-in-bud lesion is seen in left upper lobe.

**Fig 3:**
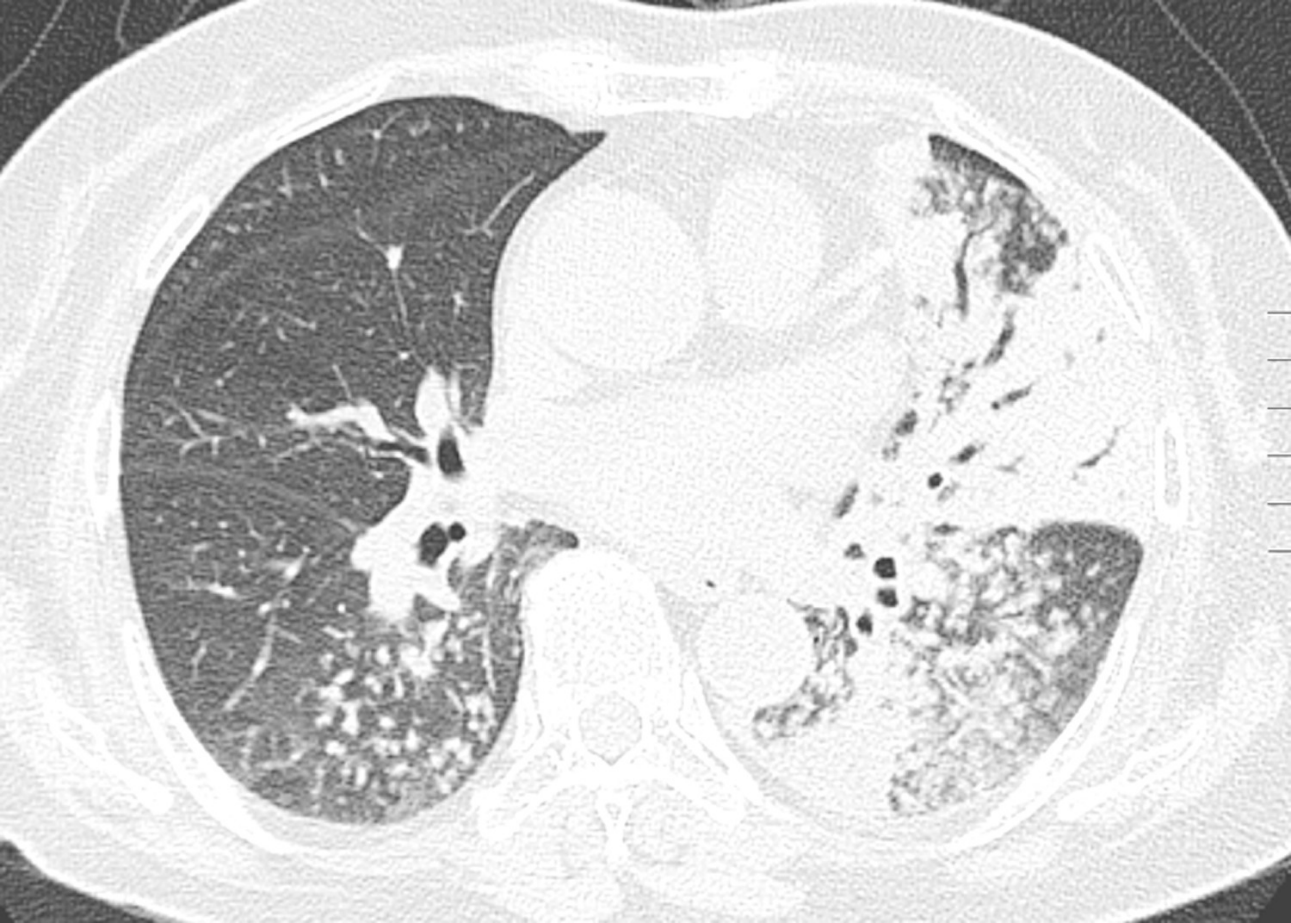
Primary multidrug-resistant tuberculosis with 58-year-old woman. Lung window setting of thin section CT shows centrilobular nodules and tree-in-bud lesions in right lower lobe, lingula and left lower lobe. Lobar or segmental consolidation and ground glass opacity are seen in lingula.

The CT findings of both primary MDR-TB and DS-TB are summarized in Table 2. Centrilobular nodules were the most common findings in patients with MDR-TB and DS-TB. Segmental or lobar consolidation, cavities in consolidation and cavitary nodules or masses were more frequently observed in patients with MDR-TB than in those with DS-TB (Figs 2 and 3). The other CT findings were not significantly different between the two groups. The PMI was significantly lower in patients with MDR-TB than in those with DS-TB.

**Table 2:** Comparison of CT findings and pectoralis muscle mass between patients with primary MDR-TB and patients with DS-TB.

CT findings	Primary MDR-TB (n = 90)	Primary DS-TB (n = 90)	p value
Centrilobular nodules and tree-in-bud	82 (91.1%)	85 (94.4%)	0.388
Large nodules	50 (55.6%)	51 (56.7%)	0.881
Lobular or subsegmental consolidation (GGO)	36 (40.0%)	32 (35.6%)	0.539
Segmental or lobar consolidation (GGO)	57 (63.3%)	32 (35.6%)	<0.001
Cavity in consolidation	32 (35.6%)	14 (15.6%)	0.002
Cavitary nodule or mass	46 (51.1%)	34 (37.8%)	0.072
Fibrotic scar	42 (46.7%)	33 (36.7%)	0.174
Bronchiectasis	29 (32.2%)	18 (20.0%)	0.062
Calcification	22 (24.4%)	26 (28.9%)	0.500
Pleural/Pericardial effusion	18 (20.0%)	18 (20.0%)	1.000
Mediastinal LNs	35 (38.9%)	27 (30.3%)	0.210
Hilar LNs	18 (20.0%)	16 (18.0%)	0.730
Calcified LNs	30 (33.3%)	23 (25.6%)	0.252
Non-calcified LNs	50 (55.6%)	38 (42.7%)	0.085
Bilateral	58 (64.4%)	35 (38.9%)	0.001
Emphysema	10 (55.6%)	8 (44.4%)	0.619
Pectoralis muscle (mm^2^)	1350.5 ± 434.2	1677.6 ± 500.2	<0.001
Pectoralis muscle index (mm^2^/kg/m^2^)	61.7 ± 16.7	79.0 ± 19.2	<0.001

DS-TB, drug sensitive tuberculosis; GGO, ground glass opacity; LNs, lymph nodes; MDR-TB, multidrug resistant tuberculosis.

Bilateral parenchymal lesions were more frequently observed in patients with MDR-TB than in those with DS-TB. The extent of total pulmonary abnormalities was significantly more severe in patients with MDR-TB than in those with DS-TB (Table 3). The extent of centrilobular nodules, segmental or lobar consolidation, cavity in consolidation, and cavitary nodule or mass was significantly more severe in patients with MDR-TB than in those with DS-TB (Table 3).

**Table 3:** Comparison of extent of lung parenchymal abnormalities in patients with primary MDR-TB and patients with DS-TB.

CT findings	Primary MDR-TBc	Primary DS-TBc	p value
Total	3.53	2.56	<0.001
Centrilobular nodules and tree-in-bud	2.88	2.38	0.068
Large nodule	0.96	0.71	0.397
Lobular or subsegmental consolidation (GGO)	0.84	0.56	0.292
Segmental or lobar consolidation (GGO)	1.10	0.64	0.001
Cavity in consolidation	0.68	0.20	0.001
Cavitary nodule or mass	0.97	0.42	0.001
Fibrotic scar	0.83	0.59	0.162
Bronchiectasis	0.52	0.28	0.073
Calcification	0.41	0.49	0.497

Six locations (five lobes and the lingular segment) were used to describe the extent of lung parenchymal lesions.

DS-TB, drug sensitive tuberculosis; GGO, ground glass opacity; MDR-TB, multidrug resistant tuberculosis.

Binary logistic regression analysis was performed to determine which variables, including patient characteristics, CT findings and PMI, were associated with MDR-TB. In univariate analyses, segmental or lobar consolidation, cavity in consolidation, cavitary nodule or mass, and bilateral involvement were significant factors for MDR-TB (Table 4). In stepwise multivariate logistic regression analysis, low PMI (odds ratio, 2.776; 95% confidence interval, 1.450–5.314; p = 0.002), segmental or lobar consolidation (odds ratio, 3.123; 95% confidence interval, 1.629–5.987; p = 0.001), and cavitary nodule or mass (odds ratio, 2.790; 95% confidence interval, 1.348–5.176; p = 0.002) were significant factors for MDR-TB (Table 5).

**Table 4:** Univariate regression analysis for risk factors of multidrug resistant tuberculosis.

Factors	B	OR	95% CI	p value
Body mass index (<21 kg/m^2^)	-0.458	0.633	0.349–1.148	0.132
Diabetes	0.197	1.217	0.598–2.478	0.588
Hypertension	-0.247	0.781	0.351–1.735	0.544
Fever	-0.100	0.905	0.377–2.173	0.823
Cough	0.074	1.077	0.506–2.292	0.847
Hemoptysis	-0.393	0.675	0.245–1.859	0.447
Dyspnea	1.043	2.836	0.966–8.325	0.058
Chest pain	-0.741	0.477	0.138–1.644	0.241
Extrapulmonary	-0.194	0.824	0.242–2.802	0.756
AFB smear positive	0.537	1.711	0.948–3.085	0.074
Pectoralis muscle index (<68.2 mm^2^/kg/m^2^)	1.046	2.845	1.554–5.207	0.001
Centrilobular nodules and tree-in-bud	-0.506	0.603	0.189–1.919	0.392
Large nodule	-0.045	0.956	0.531–1.722	0.881
Lobular or subsegmental consolidation (GGO)	0.189	1.208	0.661–2.209	0.539
Segmental to lobar consolidation (GGO)	1.141	3.131	1.704–5.752	<0.001
Cavity in consolidation	1.097	2.995	1.465–6.122	0.003
Cavitary nodule or mass	0.884	2.420	1.317–4.447	0.004
Fibrotic scar	0.413	1.511	0.833–2.743	0.174
Bronchiectasis	0.643	1.902	0.964–3.753	0.064
Calcification	-0.228	0.796	0.411–1.545	0.501
Pleural or pericardial effusion	0.000	1.000	0.482–2.076	1.000
Mediastinal LNs	0.395	1.485	0.800–2.757	0.210
Hilar LNs	0.132	1.141	0.540–2.410	0.730
Calcified LNs	-0.013	0.988	0.390–2.503	0.979
Noncalcified LNs	0.376	1.457	0.764–2.777	0.253
Bilateral	1.047	2.848	1.556–5.215	0.001

AFB. Acid- Fast Bacilli; CI, confidence interval; GGO, ground glass opacity; LNs, lymph nodes; OR, odds ratio.

**Table 5:** Multivariate regression analysis for risk factors of multidrug resistant tuberculosis.

Factors	B	OR	95% CI	p value
Pectoralis muscle index (<68.2 mm^2^/kg/m^2^)	1.021	2.776	1.450–5.314	0.002
Segmental to lobar consolidation (GGO)	1.139	3.123	1.629–5.987	0.001
Cavitary nodule or mass	1.026	2.790	1.348–5.176	0.002

CI, confidence interval; GGO, ground glass opacity; OR, odds ratio.

## Discussion

Our study is the first to demonstrate that the pectoralis muscle mass on chest CT was significantly lower in patients with primary MDR-TB than in those with DS-TB. After adjusting for clinical and CT findings, low PMI, cavitary nodule or mass and segmental or lobar consolidation were significant contributing factors to differentiating primary MDR-TB from DS-TB.

The associations between body weight and TB have been demonstrated in previous studies. Underweight (BMI<18.5 kg/m^2^) was a high-risk factor for mortality in patients with TB [16,17]. Other reports have suggested that underweight or prolonged weight loss after anti-tuberculous treatment is associated with mortality in patients with MDR-TB [12,13]. However, these studies included acquired and primary MDR-TB. In our study, pectoralis muscle mass was significantly lower in patients with primary MDR-TB than in those with DS-TB, even though the BMI was not significantly different between groups. Our findings suggest that pectoralis muscle mass is superior to BMI for differentiating primary MDR-TB from DS-TB. A previous study suggested that fat-free body mass is more closely correlated with mortality than body weight in patients with TB [18]. Previous studies have usually focused on prolonged muscle wasting in follow-up studies after anti-TB treatment [5,10,11]. These results explain why patients with acquired MDR-TB were underweight compared to those with DS-TB [12,13]. The mechanism is unclear why muscle wasting was more severe in patients with primary MDR-TB than in those with DS-TB. We speculated that the patients with primary MDR-TB had poor host immunity that led to muscle wasting. It is known that T cell-mediated immunity plays an important role in tuberculous infection [19] and malignancy. Previous reports demonstrated that the numbers of CD3^+^ and CD4^+^ T cells were significantly different between MDR-TB and DS-TB [20,21]. Another study reported that the numbers of CD3^+^, CD4^+^ and CD8^+^ T cells gradually decreased during cachexia progression in C26 tumor-bearing mice [22].

We also assessed the pulmonary features on chest CT. We found that cavitary nodules or masses and cavitary consolidation were more common and extensive in patients with MDR-TB than in those with DS-TB. Cavities in patients with MDR-TB was a frequent finding in previous studies [23–25]. It is known that cavities provide the virulent conditions for caseous tubercles and prohibit the penetration of anti-tuberculous medication in TB [26]. We found that the smear-positive rate for acid-fast bacilli in sputum was significantly higher in patients with MDR-TB than in those with DS-TB, and our result was consistent with previous reports [25,27].

Our study showed that centrilobular nodules or tree-in-bud lesions were the most common findings in both groups. However, the extent of this finding was not significantly different between the two groups, and this result was consistent with previous results [23,28,29]. Tree-in-bud lesions are the most common and earliest CT features of active pulmonary tuberculosis with bronchogenic spread [6]. This feature corresponds to intrabronchiolar caseous necrosis followed by peribronchiolar granuloma in pathologic specimens [6].

Bilateral involvement and segmental to lobar consolidation showed inconsistent results compared to previous studies on identifying MDR-TB. We found that bilateral involvement of whole lung parenchymal lesions, and segmental to lobar consolidation were more frequently observed in patients with MDR-TB than in those with DS-TB. Some reports were consistent with our results [24], but another study reported that bilateral involvement was not significantly different between the two groups [23]. The previous study suggested that if the tubercle within the cavity penetrated the airway, the tubercle bacilli can multiply and become widespread in both lungs [26]. Additionally, we found that segmental to lobar consolidation was more common and extensive in patients with MDR-TB than in those with DS-TB. Some studies were consistent with our results [24], but other studies reported that the presence and extent of segmental or lobar consolidation were not significantly different between the two groups [23,28]. Our study showed that segmental to lobar consolidation was a significant factor in multivariate analysis for MDR-TB, whereas bilateral involvement was not. It needs to be further investigated whether bilateral involvement and segmental or lobar consolidation may be helpful in differentiating MDR-TB from DS-TB or if these signs might represent a severe course of active pulmonary tuberculosis.

Our study showed that the presence of fibrotic scarring, calcifications, large nodules, bronchiectasis, pleural or pericardial effusion and lymph nodes were not significantly different between the two groups. Our results were consistent with a previous study [24]. However, another report found that bronchiectasis was more common in patients with MDR-TB, whereas calcifications, large nodules and calcified lymph nodes were more common in patients with DS-TB. In particular, calcifications, bronchiectasis and calcified lymph nodes indicate the chronicity or healing process of pulmonary tuberculosis [6,24]. These findings explain why the chronic feature of pulmonary tuberculosis is frequently found in acquired MDR-TB [28], because acquired MDR-TB is more common than primary MDR-TB [1]. More studies are needed to determine which factor is essential for differentiating primary MDR-TB from primary DS-TB.

There are some limitations in this study. First, this study was a retrospective study in a single institution, and we enrolled a relatively small number of patients with MDR-TB. This selection bias might affect our results. Second, we measured the cross-sectional area of the pectoralis muscle in a single axial image. A three-dimensional volumetric measurement may be necessary to reduce intra- or interobserver error. Third, the dietary habit or steroid intake can affect the muscle mass, but these factors were not controlled in our study. Previous studies were shown that dietary protein intake can accelerate protein synthesis in the muscle [30] and lean muscle mass was significantly lower in vegan than in non-vegan [31]. Androgenic steroid can increase the lean body mass and muscle strength [32].

In conclusion, low pectoralis muscle mass, cavitary nodule or mass and segmental to lobar consolidation were more frequently observed in patients with primary MDR-TB than in those with DS-TB. Low PMI and characteristic CT findings can help avoid ineffective first-line anti-TB medication and guide appropriate treatment.

## Abbreviations

DS-TB: drug sensitive tuberculosis
MDR-TB: multidrug resistant tuberculosis
PMI: pectoralis muscle index

## References

[1] Global Tuberculosis Programme., World Health Organization. Global tuberculosis report. Geneva, Swit- zerland: World Health Organisation; 2018

[2] Cegielski JP, McMurray DN. The relationship between malnutrition and tuberculosis: Evidence from studies in humans and experimental animals. Int J Tuberc Lung Dis. 2004;8: 286–298. 15139466

[3] Van Lettow M, Kumwenda JJ, Harries AD, Whalen CC, Taha TE, Kumwenda N, et al Malnutrition and the severity of lung disease in adults with pulmonary tuberculosis in Malawi. Int J Tuberc Lung Dis. 2004;8: 211–217. 15139450

[4] Khan A, Sterling TR, Reves R, Vernon A, Horsburgh CR. Lack of weight gain and relapse risk in a large tuberculosis treatment trial. Am J Respir Crit Care Med. 2006;174: 344–348. 10.1164/rccm.200511-1834OC 16709935

[5] Mupere E, Malone LS, Zalwango S, Okwera A, Nsereko M, Tisch DJ, et al Wasting among Uganda men with pulmonary tuberculosis is associated with linear regain in lean tissue mass during and after treatment in contrast to women with wasting who regain fat tissue mass: Prospective cohort study. BMC Infect Dis. 2014;14: 1–10. 10.1186/1471-2334-14-1 24410970 PMC3922730

[6] Im JG, Itoh H. Tree-in-bud pattern of pulmonary tuberculosis on thin-section CT: Pathological implications. Korean J Radiol. 2018;19(5):859–865. 10.3348/kjr.2018.19.5.859 30174474 PMC6082770

[7] Collins J, Noble S, Chester J, Coles B, Byrne A. The assessment and impact of sarcopenia in lung cancer: a systematic literature review. BMJ Open. 2014;4: e003697 10.1136/bmjopen-2013-003697 24384894 PMC3902311

[8] Go S-I, Park MJ, Song H-N, Kim H-G, Kang MH, Lee HR, et al Prognostic impact of sarcopenia in patients with diffuse large B-cell lymphoma treated with rituximab plus cyclophosphamide, doxorubicin, vincristine, and prednisone. J Cachexia Sarcopenia Muscle. 2016;7: 567–576. 10.1002/jcsm.12115 27104110 PMC4833756

[9] McDonald MLN, Diaz AA, Rutten E, Lutz SM, Harmouche R, San Jose Estepar R, et al Chest computed tomography-derived low fat-free mass index and mortality in COPD. Eur Respir J. 2017;50: 1–10. 10.1183/13993003.01134-2017 PMC5890424 29242259

[10] Schwenk A, Macallan DC. Tuberculosis, malnutrition and wasting. Current Opinion in Clinical Nutrition and Metabolic Care. 2000 pp. 285–291. 10.1097/00075197-200007000-00008 10929675

[11] Bacelo AC, Ramalho A, Brasil PE, Dos Santos Cople-Rodrigues C, Georg I, Paiva E, et al Nutritional supplementation is a necessary complement to dietary counseling among tuberculosis and tuberculosis-HIV patients. PLoS One. 2015;10: 1–17. 10.1371/journal.pone.0134785 26313258 PMC4551799

[12] Chung-Delgado K, Revilla-Montag A, Guillén-Bravo S, Bernabe-Ortiz A. Weight variation over time and its relevance among multidrug-resistant tuberculosis patients. Int J Infect Dis. 2014;23: 20–24. 10.1016/j.ijid.2014.01.001 24657270

[13] Podewils LJ, Holtz T, Riekstina V, Skripconoka V, Zarovska E, Kirvelaite G, et al Impact of malnutrition on clinical presentation, clinical course, and mortality in MDR-TB patients. Epidemiol Infect. 2011;139: 113–120. 10.1017/S0950268810000907 20429966

[14] Anderson DE, D’Agostino JM, Bruno AG, Demissie S, Kiel DP, Bouxsein ML. Variations of CT-based trunk muscle attenuation by age, sex, and specific muscle.: J Gerontol A Biol Sci Med Sci. 2013;68(3):317–323. 10.1093/gerona/gls168 22904095 PMC3605905

[15] McDonald ML, Diaz AA, Ross JC, et al Quantitative computed tomography measures of pectoralis muscle area and disease severity in chronic obstructive pulmonary disease. A cross-sectional study. Ann Am Thorac Soc. 2014;11(3):326–334. 10.1513/AnnalsATS.201307-229OC 24558953 PMC4028743

[16] Lai H-H, Lai Y-J, Yen Y-F. Association of Body Mass Index with Timing of Death during Tuberculosis Treatment. PLoS One. 2017;12: e0170104 10.1371/journal.pone.0170104 28085951 PMC5234803

[17] Yen Y-F, Chuang P-H, Yen M-Y, Lin S-Y, Chuang P, Yuan M-J, et al Association of Body Mass Index With Tuberculosis Mortality. Medicine. 2016;95(1): e2300 10.1097/MD.0000000000002300 26735532 PMC4706252

[18] Mupere E, Malone L, Zalwango S, Chiunda A, Okwera A, Parraga I, et al Lean Tissue Mass Wasting is Associated With Increased Risk of Mortality Among Women With Pulmonary Tuberculosis in Urban Uganda. Ann Epidemiol. 2012;22: 466–473. 10.1016/j.annepidem.2012.04.007 22575813 PMC3377556

[19] Hermans SM, van Leth F, Kiragga AN, Hoepelman AIM, Lange JMA, Manabe YC. Unrecognised tuberculosis at antiretroviral therapy initiation is associated with lower CD4+ T cell recovery. Trop Med Int Heal. 2012;17: 1527–1533. 10.1111/tmi.12001 23130871

[20] Sun ET, Xia D, Li BH, Ma J, Dong YY, Ding SS, et al Association of immune factors with drug-resistant tuberculosis: A case-control study. Med Sci Monit. 2017;23: 5330–5336. 10.12659/msm.904309 29118314 PMC5691569

[21] Geffner L, Yokobori N, Basile J, Schierloh P, Balboa L, Romero MM, et al Patients with multidrug-resistant tuberculosis display impaired Th1 responses and enhanced regulatory T-cell levels in response to an outbreak of multidrug-resistant Mycobacterium tuberculosis M and Ra strains. Infect Immun. 2009;77: 5025–5034. 10.1128/IAI.00224-09 19720756 PMC2772532

[22] Ju JE, Kim MS, Kang JH, Lee JY, Lee MS, Kim EH, et al Potential role of immunological factors in early diagnosis of cancer cachexia in C26 tumor-bearing mice. Appl Biol Chem. 2019;62 10.1186/s13765-019-0417-5

[23] Li D, He W, Chen B, Lv P. Primary multidrug-resistant tuberculosis versus drug-sensitive tuberculosis in non-HIV-infected patients: Comparisons of CT findings. PLoS One. 2017;12: 1–10. 10.1371/journal.pone.0176354 28586348 PMC5460787

[24] Yeom JA, Jeong YJ, Jeon D, Kim K Il, Kim CW, Park HK, et al Imaging findings of primary multidrug-resistant tuberculosis: A comparison with findings of drug-sensitive tuberculosis. J Comput Assist Tomogr. 2009;33: 956–960. 10.1097/RCT.0b013e31819877ab 19940667

[25] Chuchottaworn C, Thanachartwet V, Sangsayunh P, Than TZM, Sahassananda D, Surabotsophon M, et al Risk factors for multidrug-resistant tuberculosis among patients with pulmonary tuberculosis at the central chest institute of Thailand. PLoS One. 2015;10 10.1371/journal.pone.0139986 26444421 PMC4596622

[26] Grosset J. Mycobacterium tuberculosis in the Extracellular Compartment: an Underestimated Adversary. Antimicrob Agents Chemother. 2003;47: 833–836. 10.1128/aac.47.3.833-836.2003 12604509 PMC149338

[27] Metcalfe JZ, Makumbirofa S, Makamure B, Sandy C, Bara W, Mungofa S, et al Drug-resistant tuberculosis in high-risk groups, Zimbabwe. Emerg Infect Dis. 2014;20: 135–137. 10.3201/eid2001.130732 24377879 PMC3884722

[28] Chung MJ, Lee KS, Koh WJ, Kim TS, Kang EY, Kim SM, et al Drug-sensitive tuberculosis, multidrug-resistant tuberculosis, and nontuberculous mycobacterial pulmonary disease in nonAIDS adults: Comparisons of thin-section CT findings. Eur Radiol. 2006;16: 1934–1941. 10.1007/s00330-006-0174-9 16508766

[29] Cha J, Ho YL, Kyung SL, Koh WJ, O JK, Chin AY, et al Radiological findings of extensively drug-resistant pulmonary tuberculosis in non-AIDS adults: Comparisons with findings of multidrug-resistant and drug-sensitive tuberculosis. Korean J Radiol. 2009;10: 207–216. 10.3348/kjr.2009.10.3.207 19412508 PMC2672175

[30] Wolfe R. The role of dietary protein in optimizing muscle mass, function and health outcomes in older individuals. Br J Nutr. 2012;108(S2), S88–S93. 10.1017/S0007114512002590 23107552

[31] Vanacore D, Messina G, Lama S, Bitti G, Ambrosio P, Tenore G, et al Effect of restriction vegan diet's on muscle mass, oxidative status, and myocytes differentiation: A pilot study. J Cell Physiol. 2018; 233(12): 9345– 9353. 10.1002/jcp.26427 29319158

[32] Evans NA. Current Concepts in Anabolic-Androgenic Steroids. Am J Sports Med. 2004;32(2), 534–542. 10.1177/0363546503262202 14977687

